# High quality RNA isolation from ployphenol-, polysaccharide- and protein-rich tissues of lentil (*Lens culinaris*)

**DOI:** 10.1007/s13205-012-0075-3

**Published:** 2012-06-24

**Authors:** Prasanta K. Dash

**Affiliations:** NRC on Plant Biotechnology, IARI, LBS Building, PUSA Campus, New Delhi, 110012 India

**Keywords:** RNA isolation, Polysaccharide, Polyphenols, Lipids, PCR analysis, RT-PCR analysis, Gene library, Lentil, Legumes

## Abstract

Current RNA isolation methods have limitations in their ability to yield good quality and quantity of RNA from plants that have high content of phenols, polysaccharides and storage proteins. Existing methods also do not eliminate accompanying chromosomal DNA in RNA preparation that causes false positives in gene expression studies. Standard isolation technique was modified for rapid and quick extraction of RNA, and lentil tissue most appropriate to extract good quality RNA was determined. The concentration of the phenol blocker polyvinylpyrrolidone in the extraction buffer was determined, DNase I was added to eliminate chromosomal DNA and the timing of this step was optimized. RNA up to 568 μg of RNA from 1 g of tissue was isolated from four different tissues of lentil in less than half the time typically required by reported methods. The method avoids the use of toxic phenol–chloroform, hazardous guanidinium thiocyanate (GTC) and laborious CsCl ultracentrifugation. Absorbance A260/A280 ratio of 1.9 and A260/A230 ratio of 2.7 reveal RNA to be of high purity. Modified method yielded RNA that was free from contaminants and suitable for RT-PCR and cDNA library construction.

## Introduction

Lentil (*Lens culinaris* ssp. *culinaris*) is a cool-season grain legume cultivated throughout world and in Indian subcontinent providing a vital source of dietary protein in human diets and straw for animal feed. It is a diploid (2*n* = 2× = 14), annual flowering self-pollinating crop with a genome size of 4 Gbp (Kaur et al. 2011). In terms of genomic resources, lentil has relatively limited available data, in comparison to other *Fabaceae* species. A total of 9675 EST sequences and 485 GSS sequences in lentil are present in the public domain as of 17 December, 2011 as compared to 42,578 ESTs; 50,853 GSSs in chickpea (Gujaria et al. 2011) and 23914 ESTs; 89301 GSSs in pigeonpea including the whole genome sequence (Singh et al. 2011). To identify novel genes and provide the community a functional genomics resource that will facilitate research and breeding, there is a significant need to enhance lentil genomic resources. Obtaining high-quality RNA is a crucial requirement in developing genomic resources such as performing gene expression experiments, cDNA library construction, real-time PCR or microarray analyses. Theoretically, one copy of chromosomal DNA is sufficient to generate false positives in gene expression and cDNA-based studies, which makes elimination of DNA contamination from RNA extracts essential.

Standard RNA isolation techniques such as guanidinium–phenol–chloroform extraction are reliable for animal tissues, but are generally not applicable to leguminous plant materials (Baker et al. 1990) and specifically unsuitable for lentil plants. Legumes in general and lentil tissues in particular are often rich in polyphenols, polysaccharides and lipids that interfere with or degrade RNA, thus restricting its yields and deteriorating the quality. Polyphenols limit RNA extraction as they become oxidised to quinones, which covalently bind to nucleic acids (Mattheus et al. 2003). Polysaccharides display similar physicochemical properties to RNA (Azevedo et al. 2003; Sharma et al. 2003), and coprecipitate with and contaminate RNA extracts (Singh et al. 2003). Lipids associated with proteins and carbohydrates also interfere with RNA isolation protocols. If these macromolecular contaminants are not removed, they lead to erroneous estimations of RNA quantity and interfere with reverse transcription and PCR, thus compromising gene expression studies (Koonjul et al. 1999).

A rapid modified procedure of the established techniques (Chang et al. 1993; Wan and Wilkins 1994; Birtic and Kranner 2006) is described here that overcomes the above difficulties simultaneously and avoids the use of toxic phenol–chloroform and hazardous guanidinium thiocyanate. The protocol is applicable to all types of tissues ranging from root to developing grains of lentil, which are rich in protein, lipid and phenolic compounds. The isolated RNA from all the tissues is amenable for downstream applications such as PCR, RT-PCR and cDNA library construction.

## Materials and method

### Plant material, sampling and chemicals

Field grown lentil plants were sampled for root, stem and leaf tissues 45 days after sowing. Developing grains were collected at 15 days post-anthesis. All the explants were immediately snap frozen in liquid nitrogen and stored at −80 °C until further use. Mature seeds of lentil were obtained from Pulse Research Laboratory, IARI, New Delhi. Liquid nitrogen frozen explants were freeze dried to remove water so as to avoid chemical reactions during sample handling and storage (Kranner and Grill 1996). The chemicals used were of molecular biology grade and solutions were made with double-distilled water. Consumables were RNase free, plasticware and pipette tips were treated overnight with 0.1 % DEPC-treated distilled water and autoclaved. Mortars and pestles were baked overnight at 140 °C. All centrifugation steps were conducted at 10,000 rcf at 0 °C.

### RNA isolation

The plant materials were finely powdered by grinding in liquid nitrogen and to maintain uniformity 100 mg of fine powder was used as starting material. A modified borax decahydrate extraction buffer was used for the extraction of RNA from all the tissues of lentil. The buffer contained 0.2 M sodium tetraborate decahydrate, 30 mM Methylene glycol tetraacetic acid, 1 % (w/v) Sarkosyl and 1 % (w/v) sodium deoxycholate and the pH was adjusted to 9.0 by the addition of 1 M sodium hydroxide. For each tissue sample, 500 μL extraction buffer containing 6 % (w/v) polyvinylpyrrolidone (PVP) and 0.2 % (w/v) of the reductant dithiothreitol were added. The mixture was vortexed and incubated at 80 °C for 5 min. Subsequently, 1 mg of proteinase K was added to the hot solution and was incubated at 42 °C for 1 h after vortexing. To eliminate seed reserves, 50 μL 2 M potassium chloride was added and the samples were incubated on ice for 30 min followed by centrifugation at 10,000 rcf for 10 min. Approximately, 400 μL of the lower aqueous phase was transferred to a new tube. This eliminated the lipids, which are present in the upper phase. Nucleic acids were precipitated by the addition of 0.3 volumes of 120 μL of ice-cold 10 M LiCl. After adding LiCl, the suspension colour immediately turned milky white. The sample was incubated at −80 °C for 30 min and again centrifuged for 10 min at 10,000 rcf. The supernatant was discarded and the pellet was washed with 100 μL of 2 M LiCl. The pellet containing nucleic acids was re-suspended in 100 μL T_10_E_1_ buffer. An aliquot (1 μL) of the supernatant was used for the quantification by taking absorbance at *A*260 nm. To eliminate chromosomal DNA contamination, 10 μL DNase I (10 mg/ml) was added to the solution and incubated at 37 °C for 30 min. Excess DNase I and contaminating proteins were removed by potassium acetate (pH 4.8) and ethnol precipitation.

### Quantitation and integrity of total RNA

The quantity and quality of the RNA obtained were assessed spectrophotometrically at 230, 260 and 280 nm. The *A*260/280 ratio was used to detect the contamination with proteins and *A*260/230 ratio was used to check for carbohydrate contamination. Quality and integrity of isolated RNA was verified by electrophoresing RNA on 1.5 % agarose gel and staining with ethidium bromide (Sambrook et al. 1989). The bands were visualised and photographed using Gel documentation unit.

### cDNA synthesis, RT-PCR and library construction

cDNAs were synthesized using 0.5 μg total RNA extracted from lentil roots, stems, leaves and etiolated seedlings by RT-PCR using 1st strand cDNA synthesis Kit (AMV; Roche, UK). The cDNAs were PCR amplified (with expected amplicon of 360 bp) by lectin gene (NCBI Accession No AY547295) specific primers, a gene reported to encode an insecticidal protein against sap sucking insects. PCR reactions were carried out in a final volume of 25 μL reaction mixture containing 10 mM Tris–HCl (pH 8.3), 50 mM KCl, 1.5 mM MgCl_2_, 200 μM dNTP, 0.2 μM each primer (forward: 5′-GTCATAGATG CGCCCAGCAG-3′ and reverse 5′-GTTACATTC TCTTCCTCAA GTG-3′) and 0.5 U of *Taq* DNA polymerase. The temperature profile was as follows: initial denaturation at 95 °C for 4 min, 35 cycles at 94 °C for 30 s, 57 °C for 30 s, and 72 °C for 1 min followed by final extension for 7 min at 72 °C. A ‘control PCR’ was carried out with 0.5 μg of total RNA in the absence of reverse transcriptase to check for chromosomal DNA contamination.

Using 10 μg total RNA-isolated from developing seed samples, the cDNA library was constructed with SMART cDNA library construction kit (Clontech, California, USA). The library was subsequently used for screening full-length lectin gene (unpublished data).

## Result and discussion

Standard methods for RNA extraction (Chomczynski and Sacchi 1987; Salzman et al. 1999) could not be used for lentil tissues as they are rich in multiple macromolecular components. Therefore, for isolating nucleic acids from these plants, the general approach was modified so as to prevent the formation of these complexes and remove macromolecular contaminants as quickly as possible. Lowering the temperature is thought to decrease the rate of chemical reactions irrespective of exothermic or endothermic nature of reaction. Thus, we attempted to keep temperature low during RNA extraction. When the temperature was kept at 0 °C along with the addition of phenol blocker and reducing agents, the phenolic compounds did not react with nucleic acids and were precipitated with other debris after the first centrifugation and the supernatant appeared completely clear.

On the contrary, guanidinium thiocyanate based method, highly successful in the case of animal tissues, did not yield good RNA for lentil tissues. Commercially available kits utilizing magnetic particle separation technique yields high amount of poly (A)^+^ mRNA from animal cells, but did not work well with leguminous plants. The yield of RNA isolated from lentil using those kits was much lower than that obtained using the protocol described in the present study. The current protocol is a modification of the methods described earlier (Mattheus et al. 2003; Wan and Wilkins 1994). In our study, GTC has been replaced by sodium tetraborate and sarkosyl followed by LiCl precipitation.

**Fig. 1:**
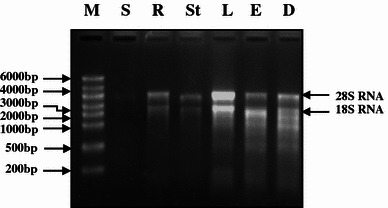
Isolation of Total RNA from different tissues of lentil. *Lane M* Riboruler High Range RNA ladder, *S* mature seed, *R* root, *St* stem, *L* leaf, *E* etiolated seedling, *D* developing seeds

When RNA was isolated by the current method, 568 μg of RNA was obtained from 1 g of leaf tissue followed by 201 μg from roots and 186 μg from stems. Etiolated seedlings yielded 58 μg RNA, while minimum of 4.7 μg RNA was obtained from developing seeds (Table 1). The *A*260/*A*280 and *A*260/*A*230 ratios of the RNA extracts were as high as 1.9 and 2.7, respectively, which indicates the presence of very low amounts of contaminating proteins, polysaccharides and polyphenol compounds. Purity of RNA samples (*A*260/*A*230 ratio) isolated from leaves was 1.9, while in case of stem tissues it was 1.7. The bands corresponding to 28S and 18S rRNA were significantly prominent in RNA extracted from leaves than other tissues indicating amenability of leaf tissues for the isolation of RNA. Gel electrophoresis of RNA samples isolated from all tissues resulted in firm and distinct bands of 28S and 18S rRNA and showed the RNA to be of excellent quality (Fig. 1; lane R–D). Since matured seeds contain high molecular weight storage proteins along with lipids and polysaccharides, we could not extract any RNA (lane S) from them.

**Table 1:** Total RNA yield isolated from different tissues of lentil using borax deca-hydrate protocol

Plant Tissues	Borax decahydrate method		
	RNA Yield (μg/g tissue)	RNA purity	
		A260/A280	A260/A230
Leaf	568 ± 41	1.9 ± 0.01	2.7 ± 0.14
Root	201 ± 23	1.73 ± 0.08	2.6 ± 0.2
Stem	186 ± 18	1.7 ± 0.11	2.4 ± 0.7
Etiolated seedling	58 ± 11	1.86 ± 0.44	2.3 ± 0.24
Developing seed	4.7 ± 1.9	1.8 ± 0.21	1.9 ± 0.1
Mature seed	0	ND	ND

Data represent mean ± SD of three replicates

*ND* Not determined

Rapid extraction of high-quality RNA from all of the tissues became possible after resolving the following key concerns: use of low temperature and optimum PVP concentration; removal of excess macromolecular admixtures such as proteins, polysaccharides and lipids and digestion of chromosomal DNA from RNA spool. The experiment was conducted using an extraction buffer with pH 9. However, when the same buffer with pH 7 was used, the yield and quality were less. Poor results were due to the presence of large amounts of contaminants in the plant tissues. In our protocol, an initial extraction in borax buffer removed most contaminants. It provided maximum RNA solubility and removed small endogenous nucleases, which reduce RNA yield and purity by nonspecific RNA aggregation (Kranner and Grill 1996; Wallace 1987).

**Fig. 2:**
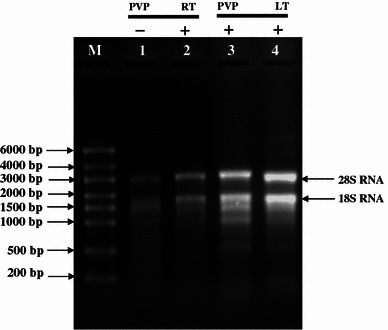
Isolation of total RNA from leaf with and without PVP and in room temperature and low temperature. *Lane M* riboruler High Range RNA ladder, *lane 1* total RNA from leaf without PVP (−), *lane 2* total RNA from leaf in room temperature (RT), *lane 3* total RNA from leaf with PVP (+), *lane 4* total RNA from leaf in low temperature (LT)

When the samples were extracted at room temperature, the supernatant appeared brown. This indicated that the phenolic compounds reacted with the nucleic acids. In contrast, when the temperature was kept at 0 °C, the phenolic compounds did not react with nucleic acids and the supernatant was clear. The low temperature not only increased the quantity of RNA but also the quality of RNA (Fig. 2; lane 2, 4) by drastically reducing the degradation of RNA. Furthermore, the degradation activity was arrested by the addition of phenol blocker polyvinylpyrrolidone. It forms complexes with polyphenols through hydrogen bonds and allows the polyphenols to be separated from nucleic acids. Similar to low temperature, we found many fold increase in RNA yield by the addition of PVP (Fig. 2; lane 1, 3). In case of xerophytic plants that contain exorbitant amount of polyphenols and many times grinding the leaves into fine powder are time-consuming, because they are hard and thick, chemically inert cross-linked form of PVP (PVPP) has been used (Wang et al. 2011). This involves sprinkling of insoluble PVPP directly onto the frozen leaf tissue in the mortar and vigorously grinding leaf tissue in the presence of liquid nitrogen to avoid the oxidation of released polyphenols into quinines, which in turn cannot bind to nucleic acids and hinder the isolation of high quality RNA. For lentil tissues, we found that the optimum concentration of PVP was 6 % (w/v) that blocked and precipitated the phenolics with other debris and the supernatant appeared completely clear after the first centrifugation. Although, some plant RNA isolation protocols prefer to use insoluble polyvinylpyrrolidone (Shukla et al. 2005), our experience with leguminous plants has shown that soluble polyvinylpyrrolidone is more effective in countering the problem arising due to the presence of phenolic compounds.

**Fig. 3:**
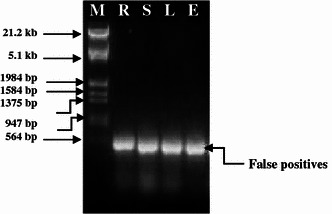
False positive PCR amplification of lectin gene using total RNA without DNase1 treatment. M = λ *Eco*R1 + *Hind*III DNA Marker, *R* root, *S* stem, *L* leaf; *E* etiolated seedling

**Fig. 4:**
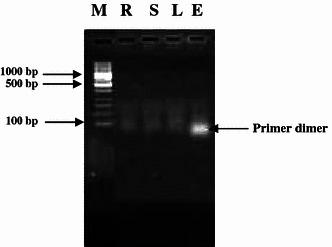
PCR amplification (showing primer dimmer) of lectin gene using DNase1 treated total RNA. M = 100 bp DNA ladder, *R* root, *S* stem, *L* leaf, *E* etiolated seedling

DNase I was applied to eliminate contaminating genomic DNA in RNA samples (Huang et al. 1996). Since the addition of DNase I at the end of the RNA isolation procedure affects the migration of RNA in the agarose gel, it was applied immediately after precipitation with lithium chloride. Excess DNase I was later deactivated and removed by acidification and centrifugation. The necessity for the use of DNase I was confirmed by running a control PCR. RNA samples, to which DNase I was not applied showed gene amplification (false positives), indicating that RNA preparation is contaminated with genomic DNA (Fig. 3; R–E). In contrast, samples treated with DNase I showed only primer dimers but no gene amplification, demonstrating that the isolated RNA was free from genomic DNA (Fig. 4; R–E).

**Fig. 5:**
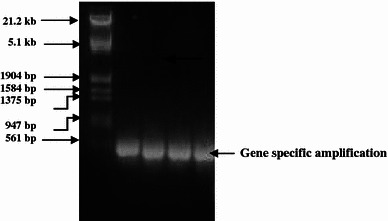
RT-PCR amplification of lectin gene using DNase 1 treated total RNA. M = λ *Eco*R1 + *Hind*III DNA Marker, *R* root, *S* stem, *L* leaf, *E* etiolated seedling

The above described method allows for the extraction of sufficient quantities of high-quality RNA in a much shorter time (4 h as compared with 18 h) than previously reported. This was achieved mainly by taking small amount of tissues and reducing the time required for the precipitation of nucleic acids with lithium chloride. A short precipitation time of 1 h resulted in an excellent quality of RNA. Overall, the described method achieved more than adequate yields for RT-PCR purposes. Therefore, reverse transcription followed by standard PCR was used to test RNA quality. After extraction of RNA from roots, stems, leaves, etiolated seedlings, cDNA was prepared by reverse transcription. Further, the single stranded cDNA was used as a template in standard PCR with gene specific primers to amplify an expected amplicon of 360 bp of lectin gene. Successful amplification of lectin amplicon (AY547295) from all RNA preparations (Fig. 5; R–E) indicates that the isolated RNA is of good quality and amenable for down stream applications such as cDNA synthesis and RT-PCR. Using total RNA isolated from leaves of lentil, a cDNA library was constructed with a titre of 3.5 × 10^6^ cfu, which is a good target library. The library was rich in full-length cDNA and we were able to isolate a full-length transcript (972 bp) through library screening approach (unpublished data).

## Conclusion

Unlike pre-existing protocols for phenolic tissues (Salzman et al. 1999; Woodhead et al. 1997; Gehrig et al. 2000), in the proposed procedure, PVP was used in combination with low temperature to circumvent the problem of phenolic compounds. In addition, no special chemicals such as GTC-based buffer or phenol–chloroform–isoamyl alcohol are necessary. This protocol is simple, fast and inexpensive to perform, allowing efficient extraction of high-quality RNA from lentil, a plant rich in macromolecular contaminants. The RNA is free of contaminating DNA and is suitable for downstream applications such as reverse transcription, gene amplification and gene library construction. The new protocol is also suitable for isolating RNA from other plant species and tissues that are devoid of phenolic compounds and thus expected to be widely applicable.

## Abbreviations

PCR: Polymerase chain reaction
RT-PCR: Reverse transcription-Polymerase chain reaction
DEPC: Diethylpyrocarbonate
KCl: Potassium chloride
LiCl: Lithium chloride
cDNA: Complimentary DNA
DNase: Deoxy-ribonuclease
μL: Microlitre
rcf: Relative centrifugal force
