# Erratum to: High quality RNA isolation from polyphenol-, polysaccharide- and protein-rich tissues of lentil (*Lens culinaris*)

**DOI:** 10.1007/s13205-013-0171-z

**Published:** 2013-09-20

**Authors:** Prasanta K. Dash

**Affiliations:** NRC on Plant Biotechnology, IARI, LBS Building, PUSA Campus, New Delhi, 110012 India

## Erratum to: 3 Biotech (2013) 3:109–114 DOI 10.1007/s13205-012-0075-3

In the article title of the original publication, the word “polyphenol” was incorrectly published as “ployphenol”. The correct article title should read as follows.

“High quality RNA isolation from polyphenol-, polysaccharide- and protein-rich tissues of lentil (*Lens culinaris*)”

